# Removal of heat-sensitive clustered damaged DNA sites is independent of double-strand break repair

**DOI:** 10.1371/journal.pone.0209594

**Published:** 2018-12-28

**Authors:** Andris Abramenkovs, Bo Stenerlöw

**Affiliations:** Department of Immunology, Genetics and Pathology, Rudbeck Laboratory, Uppsala University, Uppsala, Sweden; Tulane University Health Sciences Center, UNITED STATES

## Abstract

DNA double-strand breaks (DSBs) are the most deleterious lesions that can arise in cells after ionizing radiation or radiometric drug treatment. In addition to prompt DSBs, DSBs may also be produced during repair, evolving from a clustered DNA damaged site, which is composed of two or more distinct lesions that are located within two helical turns. A specific type of cluster damage is the heat-sensitive clustered site (HSCS), which transforms into DSBs upon treatment at elevated temperatures. The actual lesions or mechanisms that mediate the HSCS transformation into DSBs are unknown. However, there are two possibilities; either these lesions are transformed into DSBs due to DNA lesion instability, e.g., transfer of HSCS into single-strand breaks (SSBs), or they are formed due to local DNA structure instability, e.g., DNA melting, where two SSBs on opposite strands meet and transform into a DSB. The importance of these processes in living cells is not understood, but they significantly affect estimates of DSB repair capacity. In this study, we show that HSCS removal in human cells is not affected by defects in DSB repair or inhibition of DSB repair. Under conditions where rejoining of prompt DSBs was almost completely inhibited, heat-sensitive DSBs were successfully rejoined, without resulting in increased DSB levels, indicating that HSCS do not transfer into DSB in cells under physiological conditions. Furthermore, analysis by atomic force microscopy suggests that prolonged heating of chromosomal DNA can induce structural changes that facilitate transformation of HSCS into DSB. In conclusion, the HSCS do not generate additional DSBs at physiological temperatures in human cells, and the repair of HSCS is independent of DSB repair.

## Introduction

Ionizing radiation can induce damage in the DNA molecule directly or indirectly via free-radical attack [1, 2]. Both events may result in production of various types of DNA adducts and lesions, such as single-strand breaks, base modifications, abasic sites and most importantly, double-strand breaks (DSBs) [3, 4]. Prompt DSBs are immediately induced by ionizing radiation and some chemicals. These lesions are regarded as a highly toxic and they threaten the survival of cells; however, DSBs can form in cells in a controlled fashion, for example, during VDJ recombination [5]. In vivo, DSBs occur when single-strand breaks on opposite sides of the double-helix are situated within a distance of 14 bp [6]. To cope with DSBs, cells have developed several repair pathways, such as nonhomologous end joining, homologous recombination and alternative end joining (extensively reviewed in [7–9]). Essentially, these repair pathways differ in their activity throughout the cell cycle and capacity to restore the original DNA sequence at the DSB site.

In human cells, prompt DSBs are removed via fast and slow kinetics and have half-lives of approximately 7–10 minutes and 2–3 hours, respectively [10]. The repair of these DSBs is greatly influenced by the repair pathway, damage complexity and chromatin state [11–14]. Therefore, analysis of prompt DSB repair kinetics has been crucial in understanding the underlying molecular mechanisms of the repair process, DNA damage response, effect of protein deficiency and damage repair in the context of chromatin structure [10, 15].

From modeling approaches, sparsely ionizing radiation, such as X-rays, can induce clustered DNA damage sites, which represent two or more lesions in DNA that are spaced within two helical turns and induced by a single radiation track [16–18]. Further, complex prompt DSBs have been identified that contain several lesions in close proximity to the DSB ends [19, 20]. Due to the close proximity of several lesions, the damage induced in these regions can be difficult to repair [16] and, thus, the results in delayed repair. Till date, the lesions induced in clustered damage sites have not been fully characterized, and information is severely lacking regarding how these clustered sites can interact with other lesions. However, it is known that some of the lesions that are located in clustered damage sites can convert into DSBs under certain circumstances [15, 21, 22], and one such lesion is the heat-sensitive clustered site (HSCS).

Immunocytochemical assays (e.g. detection of γ-H2AX or 53BP1 foci) can provide important information of DNA damage response activation and number of DSB left unrepaired several hours after irradiation. Nevertheless, much of our knowledge about radiation-induced DSBs and their repair kinetics in mammalian cells comes from studies using constant-field or pulsed-field gel electrophoresis (PFGE). These methods often utilize cell lysis at elevated temperature and the ability of an HSCS to transform into a DSB was first identified when cells were lysed at temperatures lower than 10°C during sample preparation [22–24]. Further, it was found that heating naked DNA after exposure to ionizing radiation increased the initial DSB yield in human cells by 35–40% or more [15, 21, 22]. The HSCS are repaired within 1 hour postirradiation in all cells [10]. Several studies have discussed the biological impact of HSCS in irradiated cells [15, 21, 25], and the conclusions are conflicting. Initially, it was indicated that conversion of HSCS into DSB is merely an artifact in the analysis and that it mainly occurs when naked DNA is exposed to elevated temperatures [10, 21]. In contrast, recent results propose that HSCS may convert into DSBs in living cells [15]. Clearly, if the HSCS are transformed into DSB in intact cells, they strongly influence measurements of DSB yields, and most importantly, the estimates of repair kinetics [10, 21, 25], which in turn may have significant impact on our understanding of the major DSB repair pathways and the potential role of alternative repair pathways.

The main discrepancies of repair kinetics for different DNA extraction protocols is observed in human cells that are deficient in nonhomologous end-joining (NHEJ). When these cells are irradiated and lysed at elevated temperatures (>20°C), fast DSB repair is observed during the first hour, while no such observation can be made when cells are lysed at 4°C [10]. Thus, the results obtained from these cell lines lead to conflicting conclusions. Therefore, in this study, we investigate whether HSCS transform into DSB in live cells; additionally, we examined the potential mechanism of HSCS transformation into DSB during heating.

## Materials and methods

### Cell culture

HCT116 and HCT116 DNA-PKcs KO cells were acquired from Horizon (HD R02-049, Cambridge, United Kingdom) and grown in McCoy’s 5A medium (Biochrom KG, Darmstadt, Germany) supplemented with 2 mM L-glutamine (Biochrom KG), 100 IU/ml penicillin and streptomycin solution (GIBCO, Grand Island, NY, USA) and 9% FBS (Sigma-Aldrich, LLC, St. Louis, MO). GM5758 (Human Genetic Mutant Cell Repository, Camden, USA) cells were grown in Eagles Minimum Essential Medium that contained 9% FBS, 2 mM L-glutamine 100 IU/ml penicillin and streptomycin and vitamins. For incubation at a low repair temperature, CO_2_-independent media (GIBCO) that was supplemented with L-glutamine, FBS, penicillin and streptomycin was used.

### Pulsed-field gel electrophoresis (PFGE)

The PFGE was performed as previously described [21]. In brief, DNA of HCT116 cells were labeled for two doubling times with 2 kBq/ml of C-14 methyl-thymidine (PerkinElmer, USA), while GM5758 cells were labeled for 4–6 doubling times. Before irradiation, the cells were cooled for 20–30 minutes on ice, while the control and 0 time plugs were prepared for irradiation separately, in serum-free media. Then, cells were irradiated with 40 Gy of gamma radiation originating from a Cs-137 source (Gammacell extractor 40, MDS Nordion, Canada) at a dose rate of ~1 Gy/minute on ice. After irradiation, cold media was exchanged with warm media except for the time 0 and control samples, which were placed in lysis (ESP) buffer at pH 8.0 that contained 0.5 M EDTA (Sigma-Aldrich), 2% sarcosyl (Sigma-Aldrich) and ~1 mg/ml Proteinase K (Roche Diagnostics GmbH, Germany). Five minutes before the end of the stated repair time, the cells were washed with 1X PBS, trypsinized or scraped off the surface, mixed with low-gelling-point agarose (InCert, Lonza, USA) and placed at 4°C for 20–30 minutes, followed by transfer to ESP buffer. In experiments where lower repair temperatures were used, cells were scraped from the dish, spun down, washed with cold 1X PBS, transferred to warm agarose, immediately casted in cold molds and cooled down. Later, the ESP lysis buffer was replaced with high-salt solution (pH 7.5, 4 mM Tris (Sigma-Aldrich), 1.85 M NaCl (Merck, Germany), 0.15 M KCl (Merck) and 5 mM MgCl_2_ (Merck)) for 17–20 hours. Next, the plugs were washed in 0.5 M EDTA 3 times and incubated at 4°C (which represents prompt DSBs), 37°C-50°C (which represents HSCS and prompt DSBs) for 0–24 hours. In the repair studies, the plugs were heated at 50°C for 18–20 hours. The next day, the plugs were incubated twice in 0.1 M EDTA (pH 8.0) for 1 hour and once in 0.5X TBE for 1 hour. Next, the plugs were embedded in 4°C cold 0.8% agarose (SeaKem Gold, Lonza). DNA fragments were separated using the Gene Navigator system (Amersham Pharmacia Biotech, Uppsala, Sweden) according to the following procedure: 10 minute pulses for 3 hours, 20 minute pulses for 5 hours and 20 min, 30 minute pulses for 8 hours, 40 minute pulses for 9 hours and 20 minutes and 60 minute pulses for 20 hours at 2 V/cm in 0.5X TBE. The gel was stained with 0.5 μl/ml ethidium bromide and destained overnight in deionized water. Further DNA fragments were cut into pieces that were smaller or larger than 5.7 Mbp using *S*. *pombe* (Bio-Rad, USA) chromosomes as marker. To equalize the volume of the smallest gels, 2 ml water was added, and all samples were supplemented with 1 ml of 0.2 M HCl. Samples were heated at 95°C for 1 hour and after cooling to room temperature, 5 ml liquid scintillation fluid was added (Quicksafe A, Zinsser analytic, United Kingdom). On the following day, samples were counted using a liquid scintillation machine (WinSpectral 1414, Wallac) for 10 minutes at room temperature. In the graphs FAR<5.7 represents the ratio of relative activity in fragments that are smaller than 5.7 Mbp over the total activity in the plugs. The FAR values of heated and unheated control samples were subtracted from the corresponding treatment group’s FAR values.

### Antibody staining

GM5758 cells were plated on glass slides and treated with 1 μM NVP-BEZ235 (Cayman Chemicals, USA) 1 hour prior to irradiation with 1 Gy. The cells were allowed to repair in the presence of the inhibitor for the indicated time. Later, the slides were washed in 1X PBS, and the cells were fixed in ice-cold methanol for 20 minutes. Further, the glass slides were immersed in acetone for 10–15 seconds and air dried. Specimens were blocked in 10% FBS for 1 hour at room temperature; 53BP1 antibody (ab36823, rabbit polyclonal, Abcam, United Kingdom) and ɣH2AX antibody (05–636, mouse monoclonal, Millipore, Merck) were diluted in the ratios 1:1000 and 1:100, respectively, in 1% FBS, and the cells were incubated in this solution overnight at 4°C. The next day, glass slides were washed 3 times in 1X PBS for 5 minutes, and secondary anti-rabbit Alexa 555 or anti-mouse Alexa 488 antibodies (Invitrogen, USA) were added for 1 hour at 37°C. Next, the cells were washed 3 times in 1X PBS, followed by incubation for 5 minutes with 1 μg/ml DAPI solution. Further, the glass slides were washed in 1X PBS for 15 minutes, followed by one wash in MQ water for 5 minutes. Next, the samples were air dried, and Vectashield mounting media (Vector Laboratories Inc, USA) was added. Images were acquired using a Zeiss LSM 510 confocal microscope (Carl Zeiss, Germany), and foci were counted using ImageJ (NIH, Bethesda, MD, USA).

### Atomic force microscopy (AFM)

pBR322 plasmid was purchased from Fisher Scientific (Sweden) and irradiated at room temperature with gamma radiation in a stock solution that contained 10 mM TEN and 1 mM EDTA. Φ174 plasmid for ssDNA visualization was purchased form New England Biolabs (USA). The plasmid solution was heated in 40 mM HEPES buffer at 50°C for 3, 6 and 24 hours or kept at room temperature. The surface of mica was cleaved and pretreated with 10 mM NiCl_2_ in 40 mM HEPES for approximately 1 minute. Later, the mica was rinsed with MQ water, and 1 μg/ml DNA was applied at the respective temperature on the cleaved mica in 10 mM NiCl_2_ and 40 mM HEPES buffer. After 5 minutes, unbound DNA was washed away, and the mica surface was dried at room temperature or at 50°C. Next, the mica was stored in a closed box until analysis, when a buffer that contained 10 mM NiCl_2_ and 40 mM HEPES was added to the surface. Imaging was performed in liquid, using the peak force tapping mode. For imaging, ScanAsystFluid+ probe with 150 kHz resonance frequency and a spring constant of 0.7 N/m (Bruker, Massachusetts, USA) was mounted in a Dimension FastScan Bio system (Bruker, USA). Acquired images were analyzed using NanoScope software (v1.5, Bruker, USA).

### Plasmid gel electrophoresis

pBR322 plasmid was irradiated with gamma radiation in buffer containing 10 mM TEN and 1 mM EDTA at room temperature. After irradiation, the plasmid was diluted 1:10 with 0.5 M EDTA and heated at 50°C or stored at 4°C for the indicated time. Next, 0.5 μg of plasmid was mixed with 6X DNA loading dye (ThermoScientific) and loaded into 1% agarose gel (Sigma-Aldrich, USA). The gel was run for 3 hours at 5 V/cm, followed by staining in 0.5 μg/ml ethidium bromide (Sigma-Aldrich, USA) for 1 hour and destaining for 2 hours in deionized water. Further, plasmid conformation changes were observed using a UV illumination table and quantified using ImageJ. After analysis, the supercoiled fraction was multiplied by 1.22 to compensate for decreased uptake of ethidium bromide in the supercoiled fraction [26].

### Statistical analysis

Statistical analysis was performed using GraphPad Prism v6.07 (LaJolla, CA, USA). Comparison of two data sets was performed using two-tailed t-test where p<0.05 was considered statistically significant.

## Results

### Heat-sensitive clustered sites are repaired independent of DSB repair

**Fig 1:**
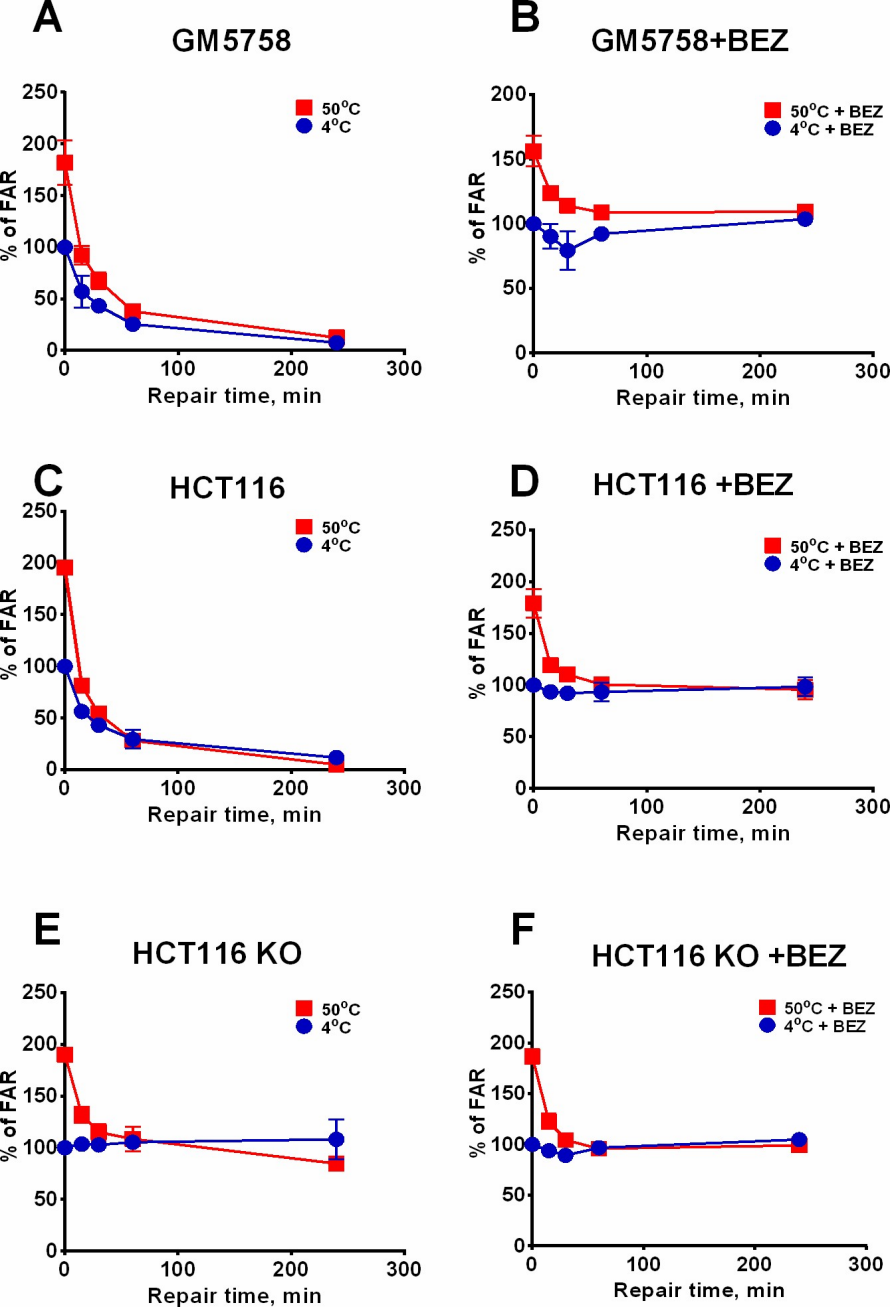
Heat-sensitive clustered sites are rejoined independent of DSB repair. Human GM5758, HCT116 and HCT116 DNA-PKcs KO cell lines were pretreated one hour prior to irradiation with 40 Gy without (A, C, E) or with (B, D, F) 1 μM NVP-BEZ235. After repair (0–4 h), chromosomal DNA was extracted and treated at 4°C or 50°C, respectively, and DSB was measured via pulsed-field gel electrophoresis. The blue lines represent the repair of prompt DSBs (samples lysed in 4°C), while the red lines represent the repair of HSCS and prompt DSBs (samples treated at 50°C). Data were normalized to 4°C samples at 0 minute repair time, and the error bars represent standard deviation data from 3–4 independent experiments.

Previously, it was observed that HSCS are transformed into DSBs under physiological conditions in nuclear extracts, suggesting that the repair proteins or histones are responsible for HSCS transformation into DSBs [15]. To test whether this process takes place in living cells, we inhibited the repair of prompt DSBs using NVP-BEZ235, an inhibitor, which has been reported to impede DNA damage response and DSB repair [27]. It was evident that NVP-BEZ235 inhibited DNA rejoining in a dose-dependent manner, and the inhibition efficiency was cell-line-dependent (S1 Fig). A lower concentration of NVP-BEZ235 was required to fully inhibit DSB repair in HCT116 cells (~600 nM) than in a normal human fibroblast cell line (800 nM). Thus, in further experiments, 1 μM NVP-BEZ235 was applied to all cell lines to ensure complete inhibition of DSB repair and to exclude variations of response in different cell lines. The kinetics of DSB repair was measured in GM5758, HCT116 and HCT116 DNA-PKcs KO cell lines and was compared with that in untreated samples (Fig 1). The two DNA-PKcs wild-type cell lines were able to fully repair DSBs within 4 hours, while no repair was detected in the DNA-PKcs KO cell line, as observed in samples treated with cold lysis buffer, which represent the repair of prompt DSBs (Fig 1A, 1C and 1E). Treatment with NVP-BEZ235 exhibited no effect on the DSB repair in DNA-PKcs KO cells (Fig 1F), while in the DNA-PK wild-type cells, NVP-BEZ235 treatment inhibited DSB repair up to 4 hours after irradiation (Fig 1B and 1D), which resulted in a repair capacity similar to that in the DNA-PKcs KO cell line (Fig 1E).

Notably, HSCS are fully removed within the first hour of repair in all cell lines (Fig 1A, 1C and 1E), and the treatment with NVP-BEZ235 did not affect the kinetics of HSCS repair (Fig 1B, 1D and 1F). Additionally, we did not detect any increase in DSBs within the first 4 hours of repair in the NVP-BEZ235-treated samples nor in the DNA-PK-deficient cell line, which indicated that the repair of HSCS does not transform these lesions into DSBs in live cells. It was observed that the -BEZ235-treated cells slightly repaired DSBs at 15 and 30 minute time points in GM5758 and HCT116 cells (Fig 1B and 1D). To assess if this repair is associated with removal of the inhibitor during the trypsinization step, HCT116 cells were pretreated for 1 hour with NVP-BEZ235 and irradiated, followed by removal of the inhibitor from the repair media (S2A Fig), or were irradiated, followed by addition of NVP-BEZ235 in the media without pretreatment (S2B Fig). These experiments showed that removal of NVP-BEZ235 partially restores the DSB repair capacity, while addition of the inhibitor rapidly inhibits the repair. Thus, this result suggests that NVP-BEZ235 can quickly cross the cell membrane, and the decrease in DSBs that was observed at early time points in the NVP-BEZ235-treated samples is a result of removal of the inhibitor during the trypsinization process (Fig 1B and 1D).

**Fig 2:**
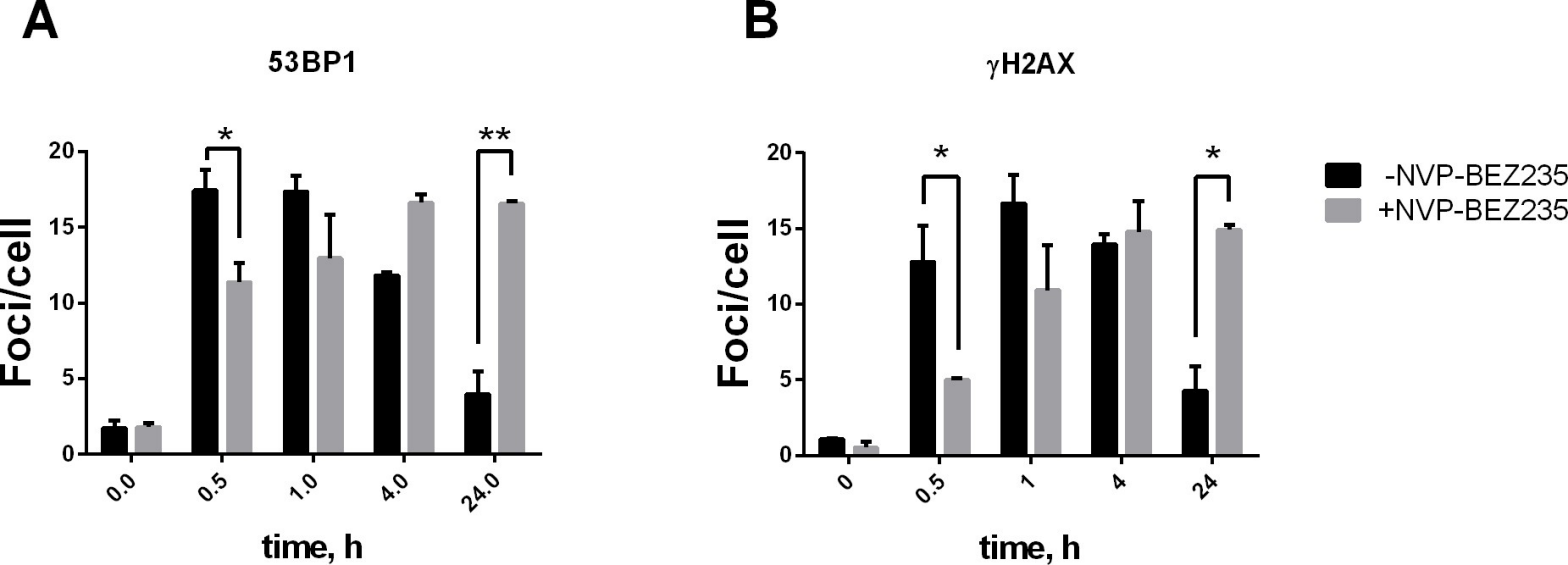
HSCS are not converted into DSB foci when DSB repair is inhibited. GM5758 cells were treated with or without 1 μM NVP-BEZ235 for 1 hour prior to irradiation with 1 Gy and allowed to repair for the indicated time. Removal of 53BP1 (A) and ɣH2AX (B) foci are shown. Error bars represent the standard error of the mean from two independent experiments, and more than 50 nuclei were scored in each experiment. *p<0.05, **p<0.01.

To further confirm that prompt DSBs are not repaired in cells treated with NVP-BEZ235, we scored 53BP1 and ɣH2AX foci over 24 hours in GM5758 cells. In these experiments, NVP-BEZ235 delayed the formation of 53BP1 and ɣH2AX foci compared to that in untreated cells (Fig 2A and 2B). The number of 53BP1 and ɣH2AX foci observed at 4 hours post irradiation did not change over a period of 24 hours in the NVP-BEZ235-treated cells, while the untreated cells almost completely removed all foci of 53BP1 and ɣH2AX (Fig 2A and 2B). Further, there was a notable delay in foci formation in the NVP-BEZ235-treated cells; however, the number of foci never exceeded the maximum levels that were observed in untreated cells. This result indicates that additional DSBs are not generated during repair, thus confirming our results from the PFGE experiments.

### Decreased repair temperatures slows HSCS repair without affecting DSB numbers

**Fig 3:**
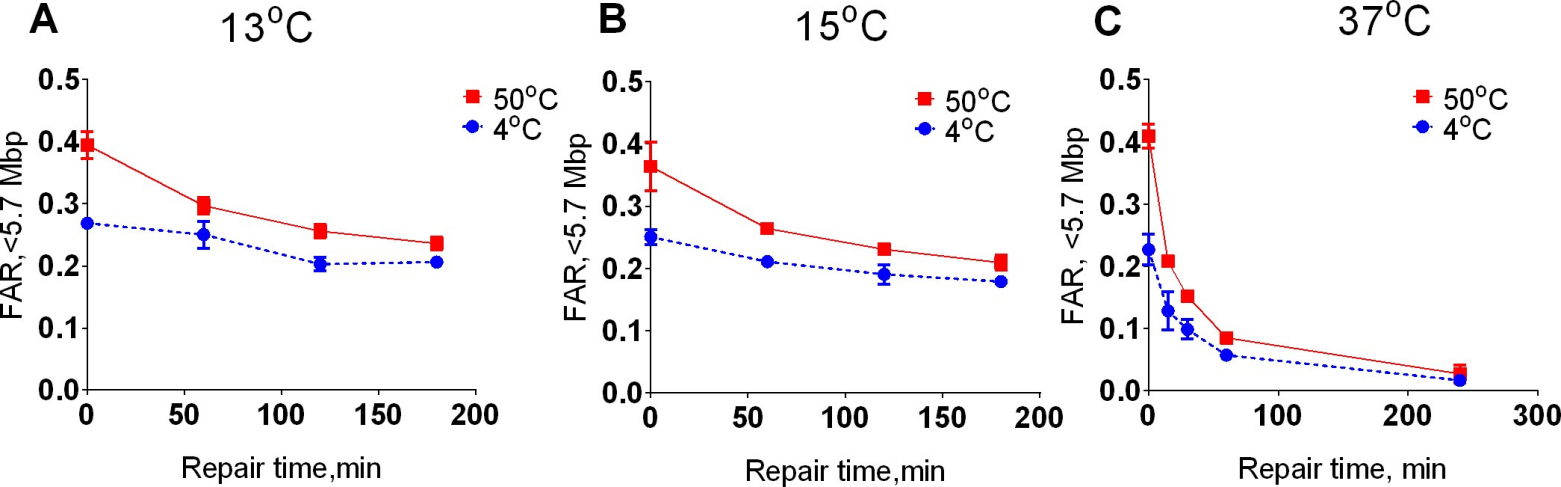
Decreased repair temperature exhibits differential effects on repair of prompt DSB and HSCS. GM5758 cells were irradiated with 40 Gy gamma radiation and allowed to repair at 13°C (A), 15°C (B) and 37°C (C) for the indicated time. Data represented as 37°C is taken from Fig 1A. After repair (0–4 h), chromosomal DNA was extracted and treated at 4°C or 50°C, and DSB was measured via pulsed-field gel electrophoresis. The blue lines represent repair of prompt DSBs (samples lysed in 4°C), while the red lines represent repair of HSCS and prompt DSBs (samples treated at 50°C). The error bars represent the standard deviation from 2–4 independent experiments.

To further test whether HSCS can transform into DSB in living cells, we inhibited DSB repair by lowering the repair temperature [28]. The results from these experiments showed that incubating cells at 13°C almost completely inhibited DSB repair during the first hour of repair (Fig 3A, p>0.22; comparing t = 0 hours and t = 1 hour), while approximately 80% of HSCS were repaired during this period. In these experiments, HSCS repair was delayed, and almost all HSCS were repaired within 3 hours instead of 1 hour, as was observed for repair at 37°C (Fig 3C). Increasing the repair temperature partially or fully restored DSB and HSCS repair (Fig 3B and 3C). Taken together, data from PFGE and foci-scoring assays (Figs 1–3), strongly suggest that HSCS are not transformed into DSBs in living cells.

### HSCS shows insignificant transformation into DSBs at physiological temperatures, and HSCS release into the DSBs is independent of repair of HSCS

**Fig 4:**
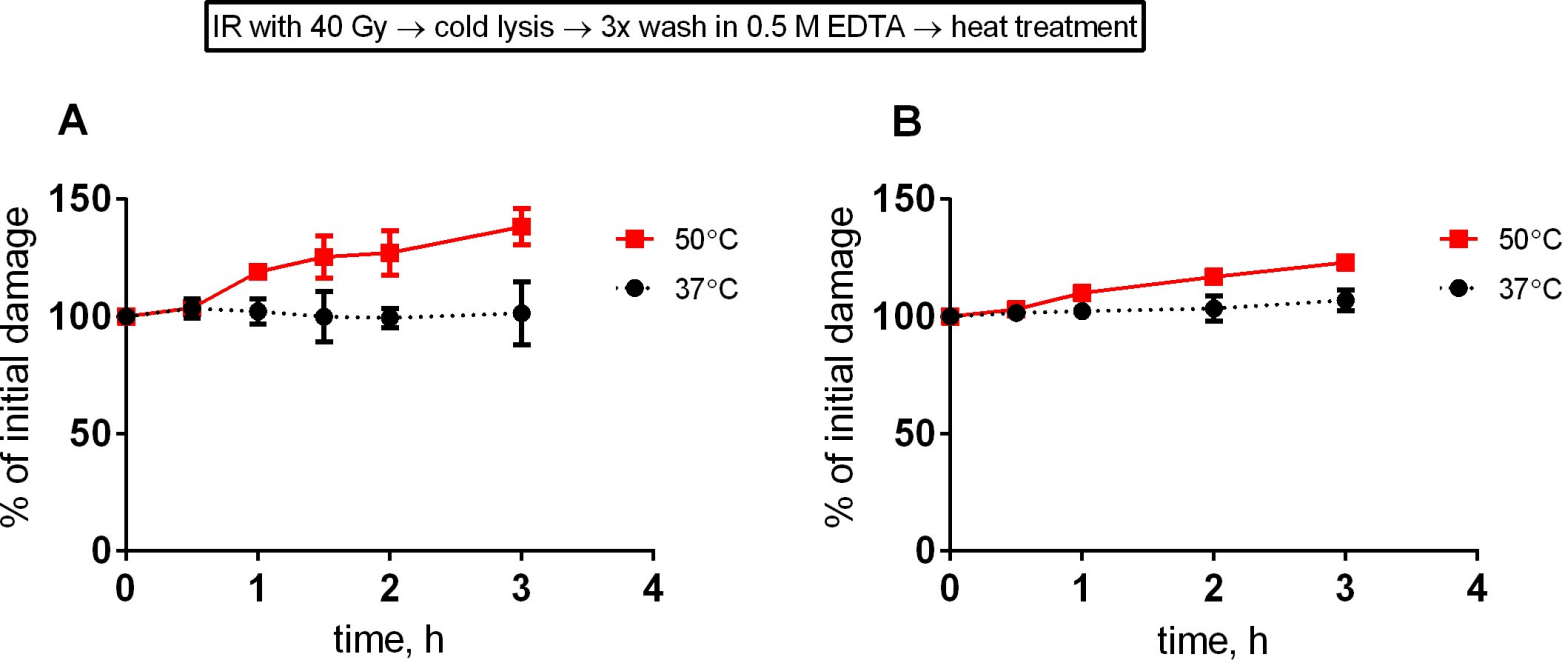
HSCS are not converted into DSB in naked chromosomal DNA at physiological temperature. HCT116 (A) and GM5758 (B) cells were irradiated with 40 Gy and lysed using the cold protocol. Next, DNA-containing plugs were washed three times in 0.5 M EDTA and heated at 37 or 50°C for the indicated time. All data points are normalized to the 0 time, and the error bars represent the standard deviation from 2–3 independent experiments.

As very little is known regarding how HSCS are released into DSBs at different temperatures at early time points, we irradiated cells and extracted DNA using the cold-lysis protocol and washed and heated naked DNA at physiological temperature (37°C) or 50°C. There was no significant transformation of HSCS into DSBs within the first 3 hours of incubation at physiological temperature in DNA that was extracted from irradiated GM5758 and HCT116 cells (p>0.05), while samples heated at 50°C started to exhibit an increased yield of DSBs (Fig 4A and 4B). Thus, we conclude that the temperature at which samples are heated plays an important role in HSCS conversion into DSBs.

### Prolonged heating of DNA induces structural changes

**Fig 5:**
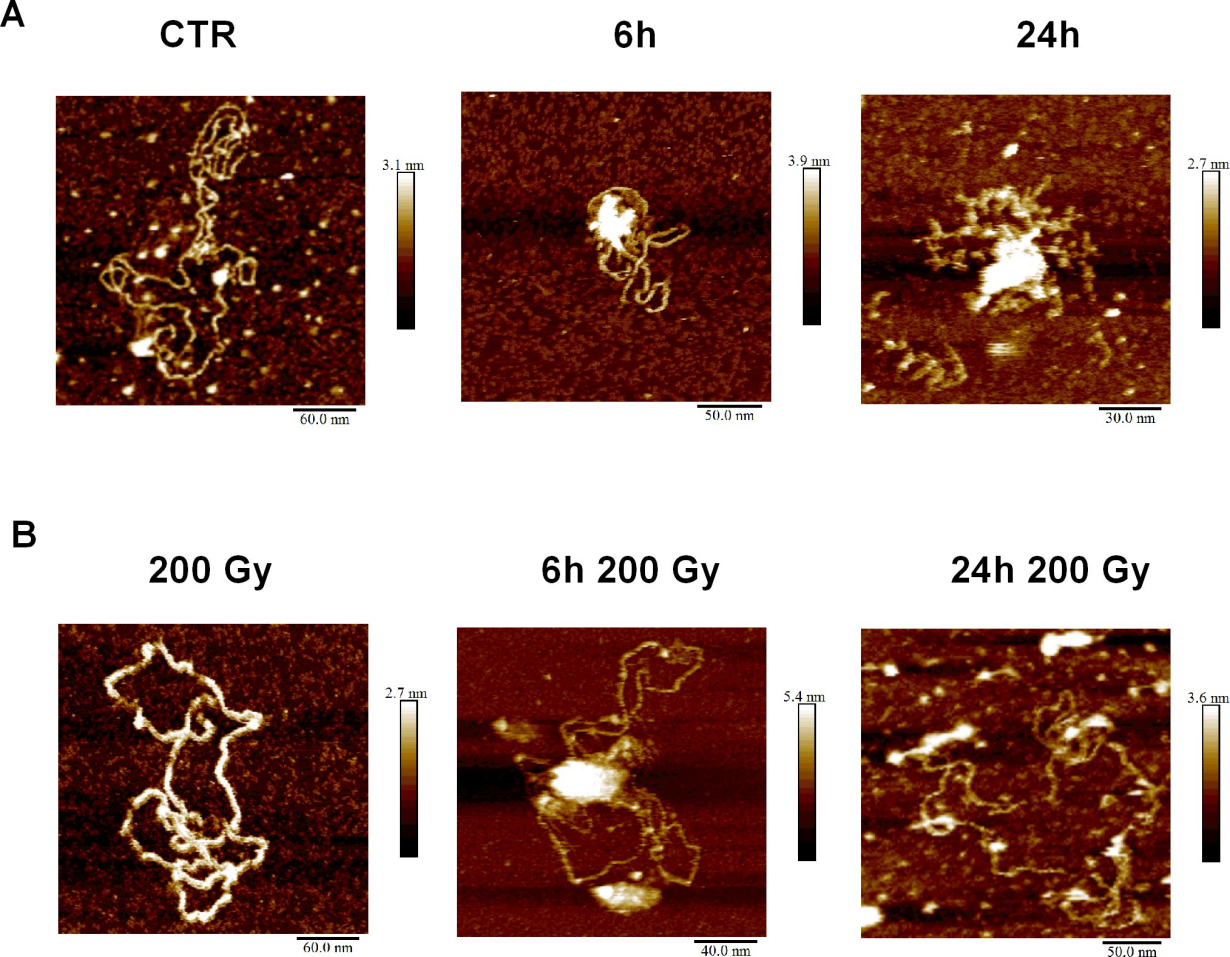
Prolonged heat-treatment induces structural changes of DNA. pBR322 plasmid DNA was irradiated with 200 Gy gamma radiation (lower panel) in 10 mM Tris and 1 mM EDTA buffer or was kept untreated at room temperature (upper panel) and heated at 50°C for the indicated time; for the control, the plasmid was kept at room temperature for 24 hours. Next, the plasmid was immobilized on mica at the indicated temperature.

**Fig 6:**
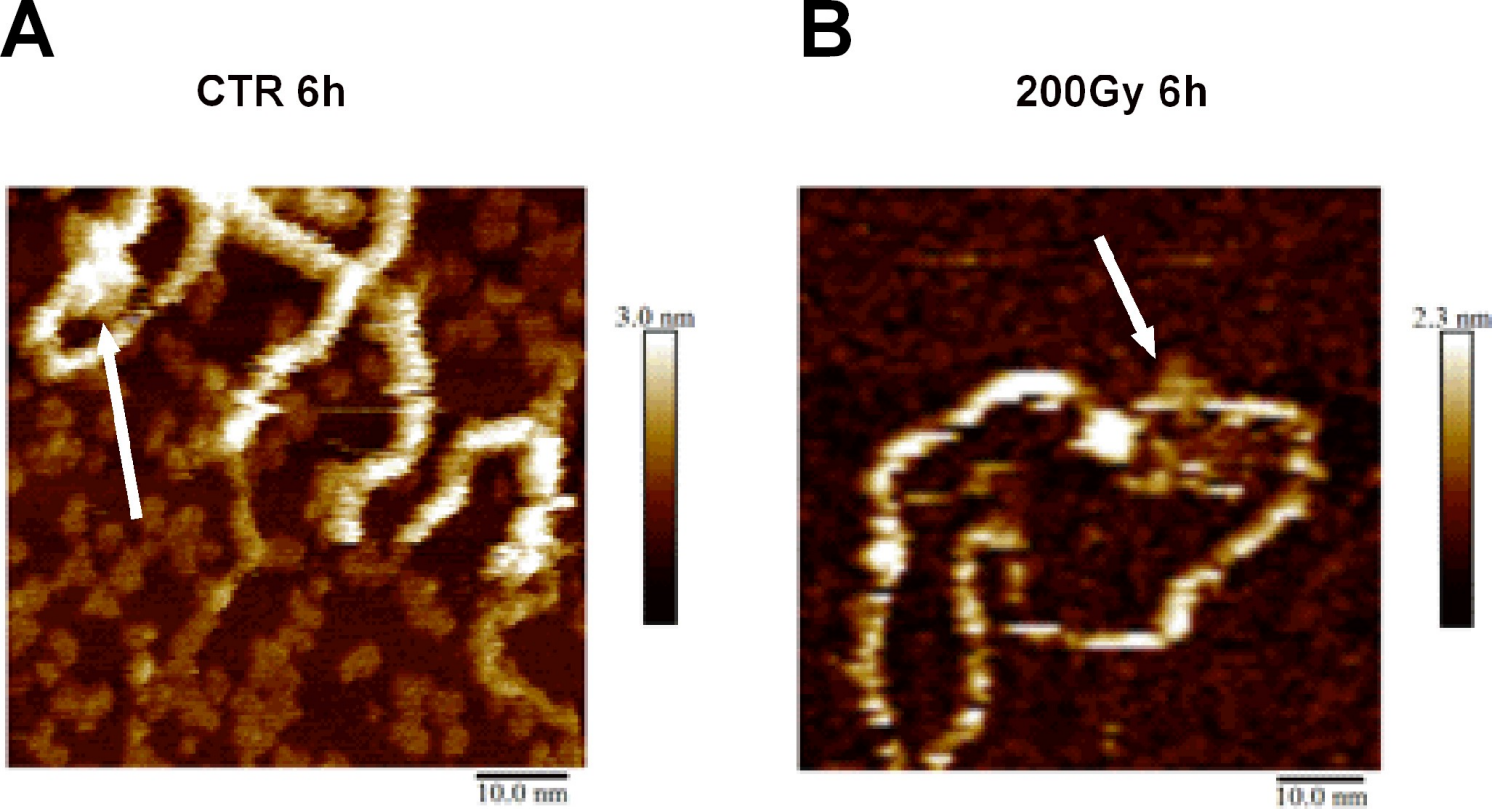
Heat-induced changes in local DNA structures. pBR322 plasmid DNA was irradiated at 0 Gy (A) or 200 Gy (B) at room temperature and heated for 6 hours at 50°C; the plasmid showed structural changes in DNA configuration. The white arrow marks the region that is consistent with DNA melting and misalignment.

To investigate if heat treatment and irradiation induce structural changes in DNA, we analyzed plasmid DNA using gel electrophoresis and atomic force microscopy (AFM). To observe how the heating time affects the structure of pBR322 plasmid (S3 Fig), we irradiated plasmid DNA at 200 Gy and 50°C. Over time, it was observed that the amount of circular fragments did not change drastically (S3A Fig), while the amount of linear fragments, corresponding to DSBs, increased (S3B Fig), and the amount of the supercoiled fraction decreased (S3C Fig). Further, we observed plasmid structural changes in AFM at the starting, intermediate and late stages of heat treatment for 0, 6 and 24 hours, respectively. Images acquired from irradiated and non-irradiated plasmids that were heated at 50°C showed aggregation of DNA (Fig 5), similar to ssDNA (S4 Fig), while no aggregation was observed for unheated plasmids (Fig 5). It was noted that the irradiated plasmid aggregates (Fig 5) were relatively larger in samples heated for 6 hours than in samples heated for 24 h. The aggregate size decreased in 24 hour heated samples, which possibly represents fragmentation of the plasmids via HSCS transformation into DSBs. Samples that were heated for 6 hours showed the formation of globular structures on DNA strands that potentially represents local melting or misalignment of DNA (Fig 6, white arrow).

## Discussion

In addition to the formation of prompt DSBs, DSB may also be formed under certain conditions after radiation exposure, e.g., during DNA repair or during postirradiation treatment at elevated pH or temperature, as observed in naked DNA. The fate of clustered DNA damage sites can vary, and it depends on the type of lesions and the proximity of these lesions [29]. However, the mechanisms that govern HSCS production, repair and their importance in live cells are not known and could also depend on the methodology used for detection. Most knowledge about DSB repair kinetics in human cells comes from studies using electrophoresis to analyze rejoining of megabase pair-sized DNA fragments. However, in the protocols used to prepare naked DNA for electrophoresis, various temperatures are applied (0–50°C), which may greatly affect the measured numbers of DSB and HSCS and, importantly, estimates of DSB repair capacity in irradiated human cells. In this study, we confirm that targeting DSB-repair pathways using NVP-BEZ235 [27] or lowering the temperature at which cells repair [28, 30] completely abolish the repair of prompt DSBs in cells. However, the inhibition of DSB repair by NVP-BEZ235 was reversible, which is in line with previous observations [31]. This could explain the decrease in DSB amount observed via PFGE at 15–30 minutes postirradiation. To exclude any potential effects produced by NVP-BEZ235 regarding HSCS release and repair, we blocked DSB repair by lowering the temperatures at which cells were incubated during repair. It has been shown that cells are unable to repair DSBs at temperatures below 13°C; however, the ability to repair SSBs, as well as other potentially less complex lesions, is retained [28]. The results that were obtained in this set of experiments indicate that cells incubated at 13°C show no significant DSB repair within the first hour, while during this time, the majority of HSCS were removed, and no additional DSBs were generated. Taken together, these experiments suggest that HSCS do not significantly contribute towards the generation of additional DSBs in cells and that HSCS repair is not dependent on the DSB repair machinery.

Additionally, we did not observe any activity with respect to alternative DSB repair pathways in DNA-PKcs-deficient or NVP-BEZ235-treated cells, which suggested that HSCS and DSB repair in human cells is independent of alternative DSB repair pathways. This observation is consistent with previous studies, which clearly demonstrated that alternative DSB repair pathways are blocked in human cells [32–34]. Furthermore, in human cells, resection of DNA overhanging ends is dependent on NHEJ factors [34], while the microhomology-mediated end joining is abrogated by factors involved in HR [35].

We have previously reported that lysing cells at physiological temperatures for extended periods of time (>24 h), leads to increase in DSBs [21]. In this study, we show that in naked DNA, HSCS are not transformed into DSBs at physiological temperatures (37°C) during 3 hours of heating. This indicates that HSCS transformation is dependent on the incubation temperature and time. Furthermore, these observations suggest that HSCS are removed before they are released into DSBs in live cells, and HSCS contribute insignificantly to the total number of DSBs under physiological conditions. In past years, it has been demonstrated that HSCS in irradiated and permeabilized cell nuclear extracts are transformed into DSBs at physiological temperatures. This implies that release of HSCS into DSBs requires the presence of proteins or a high concentration of charged amino acids in the solution [15]. These results are in contrast to our conclusion; however, we argue that permeabilized cells might not fully reveal the processes that occur in the cell nucleus in terms of proper protein-DNA interactions, repair and dynamics.

Previously, it has been shown that heating of plasmid DNA at temperatures slightly below 50°C can cause local denaturation of DNA [36], DNA heating at 55°C for 3.5 hours increases the distance at which two single-strand breaks on opposite strands can interact, and a DSB can be formed over a distance of 90–110 bp [23]. Similarly, we observed structures that are consistent with the formation of melting bubbles when plasmids are incubated in 50°C for 6 hours. Therefore, it is possible to suggest that heat exerts an extra force on the DNA structure within an HSCS, transforming it to a DSB. In light of this evidence, we conclude that heating of DNA for a prolonged time (>6 h) induces additional damage and abnormal structural changes that should be avoided during sample preparation for PFGE or other assays where primarily DSBs are measured. Thus, it is possible that heat treatment induces local melting of DNA, and HSCS are closely spaced single-strand breaks that melt at elevated temperatures and release into DSBs. An alternative explanation could be that HSCS contains heat-sensitive lesions that transform into SSBs and, subsequently, convert into DSBs. Intriguingly, we did not observe major structural changes when DNA was heated at 50°C for 3 hours (data not shown) during which 50% of HSCS are released into DSBs, suggesting that structural changes cannot fully explain the release of HSCS into DSBs. However, it appears that in live cells, such a process is unlikely to happen due to the stabilizing properties of chromatin proteins that prevent HSCS release in DSBs during the repair.

Although, the exact mechanism of HSCS transformation into DSBs still remains elusive, further studies are required to identify the exact mechanism and its contribution to cell death. It is essential to omit the release of HSCS to correctly assess DSB repair kinetics. This factor is especially important when investigating cells with deficiencies in DSB repair, inhibitor efficiency, chromatin structure repair and repair of DNA damage induced by high linear energy transfer radiation.

## Conclusions

An increasing amount of evidence suggests that HSCS are not transformed into DSBs in live cells and are uncoupled from DSBs and DSB repair. The exact mechanism or type of lesions that are responsible for the transformation of HSCS into DSBs is currently unclear. Here, we show that prolonged heating of DNA should be avoided as the heating changes the DNA structure and results in additional damage that does not form in cells under physiological conditions, thus, affecting the correct estimate of DSBs and DSB repair capacity.

## Supporting information

S1 Fig Dose response of DSB repair inhibition. GM5758 (A) and HCT116 (B) cells were exposed to increasing concentrations of NVP-BEZ 1 hour prior to irradiation with 40 Gy gamma radiation and allowed to repair for 4 hours (black bars) in the presence of the inhibitor. The FAR value at 0 time is shown in comparison as a gray bar. Error bar represents the standard deviation from two independent experiments.
(TIF)

S2 Fig DSB repair inhibition efficiency depends on the incubation conditions. HCT116 cells were preincubated with 1 μM NVP-BEZ235 and irradiated at 40 Gy, followed by removal of the inhibitor when fresh media was applied (A), or cells were irradiated at 40 Gy, followed by addition of 1 μM NVP-BEZ235 in warm media (37°C) without preincubation (B). After repair, chromosomal DNA was extracted and treated at 4°C or 50°C, respectively, and DSB was measured via pulsed-field gel electrophoresis. Error bars represent the standard deviation from 2 independent experiments.
(TIF)

S3 Fig Time- and heat-dependent changes in plasmid DNA conformation. pB322 plasmid DNA was irradiated at room temperature in 10 mM Tris-HCl and 1 mM EDTA at 200 Gy or were kept untreated and heated for the indicated time at 50°C. Data from 3 independent experiments are shown, and the error bars represent the standard deviation.
(TIF)

S4 Fig Structure of single-stranded plasmid DNA. Nonirradiated and unheated Φ174 ssDNA plasmid on the surface of mica.
(TIF)
